# Metabolomic-based promising technique for fruit-based natural products analysis

**DOI:** 10.1016/j.fochx.2025.103374

**Published:** 2025-12-06

**Authors:** Abdur Rauf, Ahmed Olatunde, Zuneera Akram, Hassan A. Hemeg, Anees Ahmed Khalil, Mohammad Ali Shariati, Zehra Edis, Muthu Thiruvengadam, Seung-Hyun Kim

**Affiliations:** a Department of Chemistry, University of Swabi, Swabi, Anbar, 94640, Khyber Pakhtunkhwa, Pakistan; b Department of Medical Biochemistry, Abubakar Tafawa Balewa University, Bauchi 740272, Nigeria; c Department of Pharmacology, Faculty of Pharmaceutical Sciences, Baqai Medical University, Karachi, 75340, Pakistan; d Department of Clinical Laboratory Sciences, College of Applied Medical Sciences, Taibah University, Madinah, 42353, Saudi Arabia; e University Institute of Diet and Nutritional Sciences, Faculty of Allied Health Sciences, The University of Lahore, Lahore, 54000, Pakistan; f Kazakh Research Institute of Processing and Food Industry, Semey Branch of the Institute, 238«G» Gagarin Ave., Almaty, 050060, Republic of Kazakhstan; g Center of Medical and Bio-Allied Health Sciences Research, Ajman University, United Arab Emirates; h Department of Crop Science, College of Sanghuh Life Science, Konkuk University, Seoul, 05029, Republic of Korea

**Keywords:** Liquid chromatography-High resolution mass spectrometry, Natural products, Bioactives, Chemical profiling

## Abstract

Fruit-based natural products have significant health and commercial benefits. Liquid chromatography-high-resolution mass spectrometry (LC-HRMS) is a powerful technique to profile complex chemical compositions. This review focuses on the use of LC-HRMS for profiling fruit-derived bioactive chemicals and demonstrates the advantages of LC for separation and HRMS for precise mass detection. LC-HRMS enables the detection of a wide range of bioactive compounds, while providing excellent precision and resolution, allowing distinction between isomeric and isobaric compounds and advancing the understanding of fruit metabolomes and their roles in food and pharmaceutical sciences. Although challenges such as complex sample preparation and limited databases persist, LC-HRMS continues to be the leading method for discovering novel bioactive compounds. Continuous improvements in analytical procedures and data resources are required to fully realize the potential of LC-HRMS. Overall, the integration of LC and HRMS has altered fruit-based analyses of natural products, providing their chemical profiles and encouraging innovations in health-related and industrial applications.

## Highlights

• LC-HRMS enables the comprehensive profiling of fruit-based bioactive compounds with high precision.
• Identification of polyphenols, flavonoids, terpenoids, and vitamins in complex fruit matrices.
• High-resolution aids in accurately distinguishing isomeric and isobaric compounds.
• The pharmaceutical and food industry applications of fruit metabolomes.
• The sample preparation and databases will enhance the analytical potential of LC-HRMS.

## 1 Introduction

Fruit-derived natural products contain bioactive compounds with various medical, nutritional, and cosmetic applications (Romero-Martínez et al., 2025). Fruits contain a variety of bioactive compounds such as flavonoids, alkaloids, phenolic acids, terpenoids, carotenoids, and anthocyanins, which contribute to their sensory qualities and health advantages (Kumar et al., 2023). These compounds have been shown to have powerful antioxidant, anti-inflammatory, anti-cancer, anti-obesity, antibacterial, neuroprotective, and cardioprotective properties (Hossain et al., 2025). As the global burden of chronic diseases increases, there is increased interest in using fruit-based bioactives to provide plant-based, sustainable solutions in functional foods, nutraceuticals, and pharmaceuticals (Morales, 2025). Despite their tremendous potential, the structural complexity and variety of fruit matrices make it difficult to isolate, identify, and quantify the bioactive compounds. Natural product mixtures frequently contain hundreds of chemicals with similar physicochemical qualities in different amounts, necessitating the use of robust, high-resolution analytical techniques (Queiroz et al., 2024). Among the recent methods, liquid chromatography combined with high-resolution mass spectrometry (LC-HRMS) has emerged as a potent platform for the in-depth investigation of complex natural product combinations, including fruit-derived products (Makni et al., 2024).

LC-HRMS has several advantages over the traditional analytical procedures. LC-HRMS combines the separation capability of liquid chromatography with the mass accuracy and precision of high-resolution mass spectrometry (HRMS), allowing for the detection of low-abundance molecules with high specificity (Anagnostopoulou et al., 2025). It is particularly suitable for both targeted and untargeted metabolomic investigations, allowing for the detailed profiling and structural elucidation of unknown compounds (Vion et al., 2025). Electrospray ionization (ESI) and atmospheric-pressure chemical ionization (APCI), which were launched in the 1980s, have transformed the integration of LC and MS by allowing the ionization of a wide range of polar, thermolabile, and non-volatile chemicals (Famiglini et al., 2021). Consequently, LC-HRMS has become an indispensable instrument for natural product chemistry, environmental monitoring, medical diagnostics, and pharmaceutical quality control. LC-HRMS has allowed for substantial breakthroughs in fruit-based natural-product research. This enables the dereplication of known compounds, elucidation of novel structures, and rapid screening of many chemical classes in a single run (Tuncay et al., 2023). Studies have shown that fruit polyphenols, flavonoid glycosides, anthocyanins, and specialized metabolites associated with health benefits outperform other methods (Zheng et al., 2025). Furthermore, LC-HRMS enables metabolite fingerprinting and chemotaxonomic classification, which are critical for discovering bioactive leads and maintaining raw material quality consistency across harvests and geographical sources (Lima et al., 2022).

LC-HRMS capabilities have increased in tandem with advancements in chromatographic materials, ion mobility methods, and machine-learning-based data processing tools. High-throughput screening, molecular networking, and automated database matching allow for real-time metabolite identification and quantification, thereby improving study reproducibility and efficiency (Celma et al., 2024). These advances have shifted natural product research from labor-intensive to data-rich precision-driven fields. The use of LC-HRMS in fruit-based natural product analysis constitutes a paradigm shift in analytical research. This provides a reliable and accurate framework for detecting the chemical diversity of fruit metabolites and linking them to their biological functions. This review examines the principles, developments, and uses of LC-HRMS in fruit-derived natural product research, emphasizing its importance as a promising analytical technique for accelerating the discovery and development of plant-based medicines and functional components.

## 2 LC-HRMS

LC-HRMS is a strong analytical platform that is frequently employed for qualitative and quantitative profiling of complex mixtures, notably in natural product research (Campmajó et al., 2023). This method combines LC and HRMS to characterize small molecules, metabolites, and bioactive substances (Núñez et al., 2024). LC separates analytes based on their interactions with the stationary phase and their solubility in the mobile phase. Reversed-phase LCs are widely used for natural product analysis because of their compatibility with a diverse spectrum of substances. Following separation, analytes are loaded into the mass spectrometer using ESI or APCI, both of which are soft ionization procedures that preserve the molecular ions (Niessen & Tinke, 1995). Ions are separated by HRMS using their mass-to-charge (m/z) ratios, which have high resolving power and mass precision. HRMS systems typically attain mass precision of 1-5 ppm, enabling precise mass determination and elemental composition prediction. Ion sources, mass analyzers, and detectors are essential components of improved software for spectral deconvolution, isotope pattern identification, and database matching, making data capture and processing easier (Neumann et al., 2014). The combination of LC with HRMS improves the sensitivity and selectivity, allowing the detection of low-abundance chemicals in complicated matrices. LC-HRMS is useful for dereplication, metabolite identification, biomarker discovery, and structural elucidation in natural-product research.

Natural product analysis is widely performed using a variety of HRMS instruments, including Orbitrap and Quadrupole Time-of-Flight (Q-TOF) mass analyzers. The Orbitrap mass analyzer works on the basis of ion trapping and oscillation in an electric field. Ultrahigh resolution (up to 1,000,000 FWHM at m/z 200) and sub-ppm mass accuracy make it suitable for untargeted metabolomics and structural interpretation (Zarrouk et al., 2022). Orbitraps are frequently combined with tandem mass spectrometry (MS/MS) to provide extensive fragmentation information for molecular identification (King et al., 2022). The Q-TOF system combines a quadrupole mass filter and time-of-flight analyzer to provide a high resolution (usually 30,000-60,000 FWHM) and fast acquisition times (Zarrouk et al., 2022). Q-TOF devices excel in data-dependent acquisition (DDA) and data-independent acquisition (DIA) procedures, providing reliable results for both targeted and untargeted studies (Chang et al., 2023). The high scan speed and sensitivity of Q-TOF make it ideal for complex plant extract profiling and bioactive chemical pharmacokinetic research (He et al., 2018). Emerging HRMS methods, such as Fourier Transform Ion Cyclotron Resonance (FT-ICR), also exhibit outstanding resolution and mass accuracy, but are less widely employed owing to their expense and complexity (Cochran & Powers, 2024). Despite these advantages, FT-ICR instruments are prohibitively expensive to acquire and operate as earlier stated because they depend on large superconducting magnets and cryogenic systems, which require specialized infrastructure and technical expertise. These high capital and maintenance costs restrict their availability to well-funded research centers and national facilities rather than routine analytical laboratories (Qi et al., 2022). Furthermore, achieving ultra-high resolution requires long transient acquisition times, reduces analytical throughput, and makes FT-ICR less suitable for high-throughput applications, such as routine metabolomics or pharmaceutical screening (Xie et al., 2022). Data processing also presents a major challenge because the extremely dense spectra produced by FT-ICR require advanced computational workflows for calibration, peak deconvolution, and molecular formula assignment (Qi et al., 2022).

**Fig 1:**
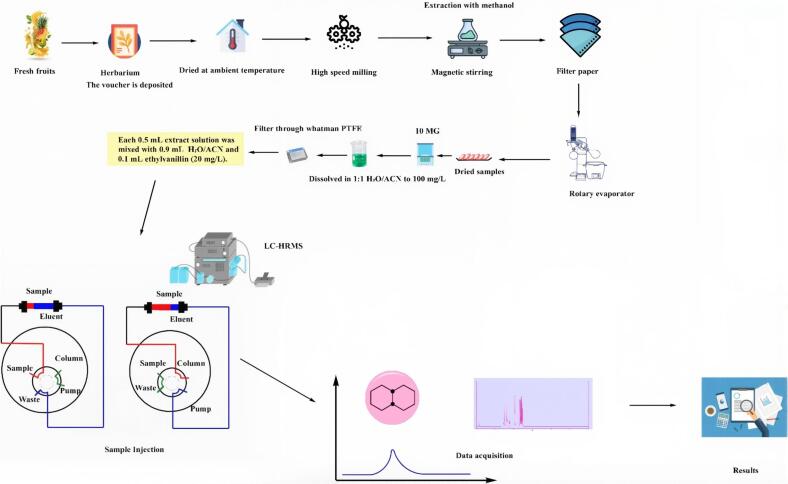
LC-MS workflow for natural products analysis (Masfria et al., 2024; Tine et al., 2017).

LC-HRMS techniques, particularly those using Orbitrap and Q-TOF devices, are critical tools for current natural product research. They allow for detailed chemical profiling and bioactivity-guided isolation, which contribute to our knowledge of complex biological systems. Fig 1 depicts the complete LC-MS analysis procedure used to profile natural products. This demonstrates the meticulously planned stages of sample preparation, complexities of data collection, and fundamental mechanism of spectrometry. This procedure sheds light on the systematic approach used to identify and characterize natural chemicals, making them suitable for use in a variety of scientific and medicinal disciplines. "Natural products" usually mean secondary metabolites created by living organisms. The small molecules (mol. wt. 2,000 atomic mass units) are not necessary for the survival, growth, development, or reproduction of an organism. Secondary metabolites from overflow metabolism owing to dietary limitations or shunt metabolism during idiophases, defensive mechanisms, or regulator molecules are generally unique to a limited subset of species within a phylogenetic group (Sarker et al., 2005). It also protects plants from herbivores and other interspecific factors. *Taxus brevifolia* and *Penicillium notatum* are taxa, and Taxol® and Penicillin G are their secondary products obtained from them.

## 3 LC–MS analysis of natural products from fruits and foods

This review explores the applications of HPLC-MS/MS and UHPLC-MS/MS for the analysis of fruit- and food-derived natural products. Natural products can also include pure compounds, such as flavonoids, coumarins, alkaloids, and other similar chemical entities (Samuelsson, 1999). The European Pharmacopoeia and the European Directorate for the Quality of Medicines (European Pharmacopoeia Commission (EPC), 2010) recommend the use of HPLC for most analytical procedures. This procedure is widely used even though the reversed phase reduces the organic phase composition (Welch et al., 2010). Instrumental analytical techniques must be universal and accessible for obtaining ecologically friendly analytical chemistry solutions. Pharmaceutical analysis followed the Reduce-Replace-Recycle (3Rs) guideline (Chen & Kord, 2009). Replacement and recycling are the main topics of study in pharmaceutical chromatographic analysis. Analytical methods have been developed by replacing harmful solvents with less toxic ones and recycling the organic solvents. During optical isomer separation, chromatographic analysis makes it difficult to minimize the organic content.

Ultra-high-performance liquid chromatography (UHPLC) and supercritical fluid chromatography (SFC) can help to implement the 3Rs rule (De Klerck et al., 2012). UHPLC, similar to conventional chromatography, proves that innovative methods are equivalent to transfer-analyte determination. Commercial UHPLC systems were first developed in 2004. Over the past decade, UHPLC has become popular for evaluating pharmaceuticals and biopharmaceuticals, demonstrating that HPLC and UHPLC can be interchanged by adjusting the gradient slope (Nováková et al., 2011). UHPLC reduces the organic solvent and analysis time without affecting sensitivity or resolution. Organic solvents limit analyte solvolysis and the exposure to dangerous chemicals in the laboratory. Green pharmaceutical analysis may benefit from UHPLC (Cielecka-Piontek et al., 2013). Separation optimization using UHPLC and HPLC adheres to the same principles, because both separation mechanisms are identical. Owing to the use of smaller particle size fills, higher mobile phase pressure values, and higher velocities, it is crucial to validate the equivalence of these techniques. According to Yang and Hodges (2005), a switch was made from a 10-minute HPLC assay to a 1-minute UHPLC assay. UHPLC and HPLC have been used to analyze pharmaceutical and biological matrices. UHPLC and HPLC with BEHC18/HSST3 and X-bridge C18/Atlantis C18 columns were used to separate folic acid derivatives. UHPLC has a higher sensitivity, linearity, and separation efficiency. The analysis runtime decreased fourfold and the LOD values decreased (Goodwin et al., 2007). In both the methods, gradient elution was required to detect a common anticancer drug in the presence of 14 contaminants in the pharmaceutical matrix, and comparable validation parameters were obtained. UHPLC analysis requires 34 min, whereas HPLC requires 90 min (Nanduri et al., 2012). Using Deming Regression analysis, Xu et al. (2009) compared UHPLC and HPLC methods to determine the concentration of the anticancer drug busulfan in human plasma. Both met the analytical instrument standards for pharmacokinetic studies (precision, repeatability, and reliability). UHPLC required 1.3 minutes compared to 10 min for HPLC. The derivatization and extraction of plasma busulfan followed the same procedure.

Researchers who use LC-MS have mostly used HPLC, whereas those who use LC-HRMS have mostly used UHPLC. Both methods used C18 columns and their commercially improved versions as fixed phases. However, in LC-HRMS, the use of superficially porous particles instead of the more common fully porous particles has increased significantly. For eluents A and B, formic acid–water and formic acid–acetonitrile solutions were the most important solvents for the mobile phase. ESI and H-ESI are the best MS ionization methods. MS should be used in the negative mode, whereas HRMS can be used in both negative and positive modes (Ruiz Gatell, 2022). Most innovative drug development assays use HPLC-MS, HPLC-MS-MS, and UHPLC-MS as an analytical method (Ackermann et al., 2002). The investigation of natural products necessitates the identification of secondary metabolites, which are highly desirable but inherently scarce (Krug & Müller, 2014). Untargeted methods are new to mass-spectrometry-driven natural product discovery (Klitgaard et al., 2014), and focus on identifying and quantifying known chemicals using LC-MS and MS/MS data. Compared to other platform technologies in the field of -omics, the development of secondary metabolomic data mining and computational methods for automated MS/MS spectral analysis is ongoing.

LC-MS is appropriate for the analysis of food samples (Di Stefano et al., 2012). The chromatography-mass spectrometry combination screen confirmed and quantified hundreds of components in a single assay. Thus, UHPLC–MS is better than traditional HPLC–MS for identifying and quantifying polyphenols in complex matrices such as plant foods, dietary constituents, and designed meals. LC-MS or LC-MS/MS is the best method for evaluating the structural characteristics of polyphenols in dietary samples, regardless of their molecular weights (Motilva et al., 2013. Flamini (2013) used liquid chromatography-mass spectrometry (LC-MS) to characterise wine polyphenolic derivatives and link them to sensory qualities, specifically grape extract polyphenols and anthocyanins. It has been shown that combining MS techniques can help characterize the various low- and high-molecular-weight polyphenols found in grapes and wine, as well as to investigate grape metabolomics (Flamini, 2013). Di Stefano et al. (2012) investigated the application of LC-MS for food analysis of various products, including milk, cheese, cereals, ham, olive oil, and wine. This study examined the common areas of application for food analysis. Finally, gastronomic authenticity, adulteration, and future orientation were evaluated (Di Stefano et al., 2012). Hoffmann et al. (2014) used liquid chromatography-mass spectrometry (LC-MS/MS) to identify and characterize natural microbial compounds. Calvaruso et al. (2020) detected 165 pesticides in citrus fruits using LC-MS/MS.

Kang et al. (2023) used liquid chromatography-mass spectrometry (LC-MS) to identify fresh and damaged beverages. Soraya et al. (2022) identified compounds in the fruit juice of Indian jujube (*Ziziphus mauritiana* L.) using LC-MS/MS. Using liquid chromatography-mass spectrometry (LC-MS/MS) and gas chromatography-mass spectrometry (GC-MS), Aslantas et al. (2023). Yajima and Okoshi (2022) used metabolome analysis and LC-MS measurements to ascertain the odor of off-broccoli despite volatile and nonvolatile chemicals. The Indian frankincense (*Boswellia serrata* Roxb. ex Colebr.) is a common element in supplements used for treating osteoarthritis and inflammatory illnesses (Trivedi et al., 2023). Mulberries are well known worldwide for their flavor, nutritional value, and medicinal properties. Jiang et al. (2023) investigated the quality of mulberry fruit over three maturities. Specific metabolomic LC-MS detected 981 compounds from 12 classes in mulberry (*Morus* spp.) fruits at three developmental phases (Jiang et al., 2023). The mediterranean region benefits from the bioactive chemical powerhouse of the carob tree (*Ceratonia siliqua* L.). The fruit of *C. siliqua* yields flour, coffee, baked goods, nectar, powder, and beverages. In this study, we optimized *C. siliqua* preparation for targeted LC-MS/MS metabolomic analysis by Deda et al. (2023). This research suggests standardizing the preparation of metabolomic samples for *C. siliqua* LC-MS/MS analysis (Deda et al., 2023).

As bioanalytical method development necessitates the quantification of medicinally active agents in biological samples, the sensitivity and selectivity of UHPLC at low detection levels generates precise, dependable data that can be used for pharmacokinetics, toxicity, and bioequivalence studies. The methodologies used for bioanalysis emphasize the importance of sample preparation. UHPLC-MS is an important tool in metabolomics and proteomics (Desai et al., 2012). Protein precipitation, solid-phase extraction, and liquid-liquid extraction are only a few of the sample preparation methods used in analytical chemistry (Kole et al., 2011). Protein precipitation, liquid-liquid extraction, and solid-phase extraction are some of the most commonly used sample preparation methods in analytical chemistry. Protein precipitation and liquid–liquid extraction are commonly employed because of their simplicity and cost-effectiveness. In contrast, solid-phase extraction (SPE) offers higher selectivity and cleaner extraction, making it suitable for a broad range of applications. SPE has been successfully applied in the quantification and analysis of various drug classes, including antibiotics (Chahbouni et al., 2015; Hillewaert et al., 2015), antioxidants (Zongo et al., 2023), antivirals (Li et al., 2016a), anticancer agents (Zhao et al., 2016), central nervous system drugs (Junnotula & Licea-Perez, 2013), cardiovascular and anticoagulant agents (Al-Dirbashi et al., 2016; Radwan et al., 2012), antifungals (Wang et al., 2015), analgesics (Hillewaert et al., 2015), steroids (Liu et al., 2015), NSAIDs (He et al., 2016), antidiarrheals (Mady et al., 2023), antidiabetic (He et al., 2016), antidepressant (Gu et al., 2015), antiepileptic (Karinen et al., 2015), proton pump inhibitors (Chunduri & Dannana, 2016), vitamins (Jacobs et al., 2016), anti-tubercular (Baietto et al., 2015), immunosuppressants (Upadhyay et al., 2014), antibacterials (Mula et al., 2023), veterinary drugs (Zhu et al., 2023), expectorants (Wen et al., 2015), and multicomponent drug analyses (Li et al., 2016b). Motilva et al. (2013) demonstrated the use of UHPLC–MS for the analysis of food polyphenols, while Lucci et al. (2017) further characterized these compounds using LC–MS and LC–HRMS.

## 4 Targeted and untargeted metabolomics approaches in fruit analysis

Metabolomics has emerged as an effective approach for analyzing the complex chemical composition of fruits and revealing information about their nutritional, physiological, and therapeutic properties (Kortesniemi et al., 2023). Metabolomic research is dominated by two basic strategies: targeted and untargeted. Each method offers unique objectives, advantages, and data interpretation methodologies essential in fruit research, such as biomarker development and quality control. Targeted metabolomics quantifies a predefined group of metabolites, generally employing high-resolution analytical technologies such as LC-MS/MS or GC-MS in multiple reaction monitoring (MRM) mode. It is frequently hypothesis driven and demonstrates high sensitivity, specificity, and quantification accuracy. This method is useful for analyzing fruit quality and nutritional composition, as well as for confirming recognized biomarkers in grapes, berries, and citrus fruits (Roberts et al., 2012). On the other hand, untargeted metabolomics aims to obtain a complete profile of all detectable metabolites in a sample without prior information. This exploratory approach employs current platforms, such as UPLC-QTOF-MS, Orbitrap, and NMR spectroscopy, to capture a broad range of metabolites. Untargeted data analysis requires complex preprocessing steps, such as peak identification, alignment, normalization, and multivariate statistical modelling, including principal component analysis (PCA) and orthogonal partial least squares discriminant analysis (OPLS-DA) (Rivera-Pérez et al., 2021). Although untargeted approaches provide broad insights, they usually encounter issues with metabolite identification, annotation accuracy, and repeatability of quantification (Jeppesen & Powers, 2023).

ANOVA or t-tests were used for statistical analysis to determine the level of significance in metabolite concentrations between treatments, fruit types, etc. (Bujak et al., 2016). When the goal is marker discovery or classification, and the targets are large, supervised multivariate techniques such as partial least squares discriminant analysis (PLS-DA) and OPLS-DA are used instead of univariate tests (Blasco et al., 2015). Additionally, in connection with annotation and pathways, the targeted metabolites were mapped to different databases such as metabolite and chemical entity database (METLIN), the human metabolome database (HMDB), kyoto encyclopedia of genes and genomes (KEGG), global natural products social molecular networking (GNPS) spectral libraries, and MassBank to identify metabolites, map pathways, and annotate the chemical class of compounds (Dadwal et al., 2022; Peng et al., 2025; Salem et al., 2020; Tu et al., 2025; Wang et al., 2024b). To reliably label metabolites and analyze pathways, both targeted and untargeted techniques use robust databases (such as METLIN, HMDB, and MassBank) and computational tools (Zhou et al., 2022).

Untargeted metabolomics has resulted in the identification of novel biomarkers that are specific to fruits and cultivars. For example, metabolomic screening of apple cultivars identified significantly differentiating metabolites linked to flavor and storage qualities (Chen et al., 2025). Similarly, metabolomic analyses of wild and cultivated chilli peppers have revealed chemicals linked to domestication (Cervantes-Hernández et al., 2022). However, targeted approaches are commonly used in quality control settings because of their high reproducibility and quantitative precision. For example, evaluating anthocyanins in blueberries or flavanones in citrus fruits can help determine ripeness, postharvest degradation, and product authenticity (Yan et al., 2023). Metabolomics has been used in grape and wine analyses to identify specific aroma compounds and polyphenols that are significant for varietal identity and sensory quality (Pinu, 2018). The combination of these approaches in a hybrid methodology that incorporates both untargeted and targeted analytical techniques provides a comprehensive framework for fruit metabolomics investigations. This dual technique improves metabolite identification accuracy, while also supporting translational research, such as the development of functional ingredients and fruit trait improvement via genetic breeding, a process that selects or modifies genes for desirable characteristics.

Recently, LC-HRMS metabolomic investigations have been found to be useful in unveiling various new biomarkers that are considered responsible for varietal differences, post-harvest quality changes, and progression of the ripening process in fruits. According to an untargeted metabolomic study conducted by Afifi et al. (2023), 11 new metabolites were identified for the first time in sweet orange peel using nLC-ESI-qTOF-MS combined with principal component analysis and OPLS-DA. Similarly, another UPLC-ESI-QTOF-MS-based untargeted metabolomics study combined with PCA/PLS-DA revealed the identification of four (2-hydroxybenzoic acid β-d-glucoside, 2-hydroxy-5-methoxy benzoic acid, pteroyl-D-glutamic acid, and pyroglutamic acid) processing markers in strawberry purees, whereas three (lysoPE(18,3/0,0), caffeic acid, and dihydroxycinnamic acid glucuronide) were identified in apple purees (Salazar-Orbea et al., 2022). Two isomers of hydroxycinnamic acid, 4-p-coumaroylquinic acid and trans-4-caffeoylquinic acid, were identified as biomarkers that discriminated between resistant and susceptible apple cultivars based on UHPLC-QTOF HRMS profiling combined with PLS-DA/OPLS-DA statistical modelling (Alonso-Salces et al., 2022). Numerous other studies have highlighted the potential of metabolomics-based investigations as a critical analytical technique for identifying novel biomarkers and authenticating fruit-based natural compounds for nutritional, functional, and quality assessments (Aung et al., 2022; Gao et al., 2024; Mostafa et al., 2023; Oak et al., 2019).

## 5 Chemical profiling of fruit-based natural products

Chemometric analysis, which involves applying statistical and mathematical methods to interpret complex chemical data when combined with HPLC, serves as a powerful approach for identifying variations among samples. This combined method enables the detection of differences in product quality, genotype, regional origin, and cultivation type (e.g., conventional brewing processes or roasting degrees). For example, HPLC can generate detailed chromatographic profiles that can be used to distinguish samples based on their chemical compositions (Klikarová & Česlová, 2022). Analytical approaches combined with supervised and unsupervised chemometrics can be used for traceability, authenticity, and bioactive profiles of fruit- and fruit-based products (Arslan et al., 2025). Hierarchical cluster analysis (HCA), linear discriminant analysis (LDA), support vector machine (SVM), principal component analysis (PCA), and partial least squares discriminant analysis (PLS-DA) are the most popular chemometric methods for tracing and authenticating fruit-based and fruit-based products (Arslan et al., 2025). This review highlights the modern knowledge on HPLC methods, which are either non-targeted or targeted, as well as chemometric investigations of fruits. In targeted screening or chemical profiling, targets are molecules with standard chemical structures and names for which quantitative targeted approaches are available, along with certain risk and exposure determination information (Smolders et al., 2009). Certain variable preparations are employed, such that the targeted molecules show the highest removal of matrix interference (Yusa et al., 2015). Authentication and estimation are usually carried out by employing low-resolution mass spectrometers, such as triple quadrupoles, which are normally controlled in certain reaction-regulating acquisition states to produce both maximum sensitivity and specificity. Supporting detection by comparing the obtained reference values with standards, including the retention time in chromatography, MS, and MS/MS spectra, was employed to standardize the molecular identity before investigation. The estimation was performed by applying the isotopic dilution approach, which allows the attainment of the highest performance with decreased uncertainty (Pourchet et al., 2020). Several international regulations, such as the EU 2002/657/EC, Codex Alimentarius guidelines, and FDA standards, provide frameworks for the validation and performance assessment of analytical methods to ensure food safety and reliability of testing results. Targeted profiling can be carried out by applying high-resolution instrumentation (for instance, flight time or Orbitrap), enabling targeted studies that are simultaneous with non-targeted and suspected studies, although some setbacks can be observed in this situation when associated with completely designed targeted procedures dependent on tandem mass spectrometry (Cajka & Fiehn, 2016).

Nontargeted chemical profiling studies have been used to analyze HPLC fingerprints and other profiling techniques, including gas chromatography linked to mass spectrometry (Toci et al., 2018; Núñez et al., 2021; Núñez et al., 2024; Abdelwareth et al., 2021), UV/VIS spectroscopy (Marcheafave et al., 2021), nuclear magnetic resonance (NMR) (Toci et al., 2018), and plasma optical emission spectrometry (Anderson & Smith, 2002), all of which are usually linked to multidimensional statistical procedures, including factor analysis (FA), discriminant analysis (DA), partial least squares regression (PLS), and PCA. Approaches to chromatographic fingerprint methods that are non-targeted are linked to documenting instrumental signals as a retention time function but without knowing any additional data, quantification, or identification of the molecules resulting in these signals. Non-complex sample processing methods are typically used to obtain several plant-based molecules (Núñez et al., 2021). Hence, non-targeted procedures demonstrate a non-complex, fast, and less expensive approach that can be used to confirm the quality and authenticity of plants and their parts such as fruits.

Although LC-HRMS is the main analytical technique employed for the characterization of non-volatile and polar metabolites present in fruits, it remains an important tool for chemical profiling of volatile and low molecular weight fractions of metabolites, which are mainly responsible for the flavor and aroma of fruits. To achieve holistic profiling, the scientific community uses GC-MS and LC-HRMS as complementary techniques, as GC-MS targets mainly fatty acids and volatile esters, while LC-HRMS targets fruit secondary compounds such as phenolic acids and flavonoids. Numerous studies have demonstrated the synergistic use of GC-MS and LC-HRMS combined with PCA and HCA (Afifi et al., 2023; Aung et al., 2022). In a recent study, chemical analysis of infected *Citrullus lanatus* (watermelon), as well as a healthy one, was performed using GC-MS. The volatile organic molecules in the fruit and seed of *C. lanatus* that is healthy and infected *C. lanatus* were analyzed using headspace-solid phase microextraction (HS-SPME), GC-MS, HCA, chemometric studies, and PCA. The findings from the aforementioned strategies can be used together with the GC-MS method to differentiate infected fruits from healthy fruits. Identification of certain phenolic molecules (p-cresol, phenol, 2-methoxy-phenol, and benzyl alcohol), 2,3-butanediol, and acetoin was observed in adulterated fruits, indicating the existence of a potential defense mechanism against infection (de Assis et al., 2023). According to a study conducted by Salazar-Orbea et al. (2022), GC-MS was employed for the detection of esters and aldehydes, whereas LC-HRMS was more targeted towards phenolic derivatives, characterized as biomarkers responsible for postharvest storage and ripening (Salazar-Orbea et al., 2022).

Multi-chemical profiling of strawberries was performed to determine the effects of cultivar and cultivation conditions using HPLC. Multi-chemical characterization was performed on the fruits of five varieties of strawberries cultivated in two cultivation systems, closed and open soilless systems, in two campaigns under various climatic conditions (i.e., temperature and rainfall). Phenolics, sugars, and essential and nonessential elements were identified in this study. In addition, different complementary statistical strategies were used to select the chemical descriptors of the agronomic and cultivar conditions. Malate and phenolic acids, including ferulic acid, caffeic acid, ellagic acid, and *p*-coumaric acid; anthocyanins, including cyanidin-3-glucoside, pelargonidin acetate, pelargonidin-3-rutinoside, pelargonidin-3-glucoside, pelargonidin derivatives 1 and 2; and sucrose were the most discriminant factors among cultivars, whereas climatic factors and the cultivation system had less effect on polyphenol content. The authors concluded that the above results demonstrate a combination of multi-chemical strategies and sophisticated chemometric tools for food traceability studies (González-Domínguez et al., 2020).

**Fig 2:**
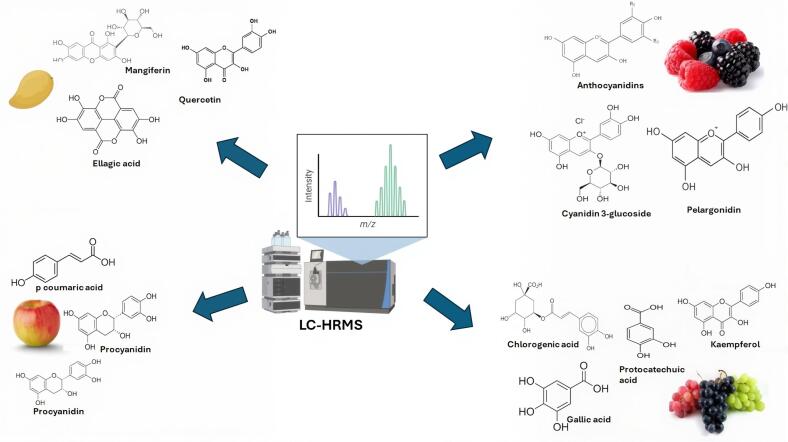
LC-HRMS Applications for detection of bioactive compounds in some selected fruits (D'Urso et al., 2020; Kukula-Koch et al., 2013).

LC-HRMS has been used to enhance the structural annotation of isomeric flavanones in citrus fruits. This study aimed to improve the annotation of molecular structures by investigating four pairs of flavanone-7-O- isomers of diglucosides and their primary aglycones, which are frequently found in citrus products. Analysis was conducted using LC-HRMS coupled with MS/MS. The research findings validated the effectiveness of this approach for biological variables, as evidenced by the relative standard deviation of the ion ratio, which represents the proportion of specific ions measured in the samples, ranging from 3.91% to 12.28%. Different fragmentation patterns of citrus flavanones were observed. The MS/MS segments of (S)-isosakuranetin and (S)-hesperetin were complex and indicated typical radical ions [^1,2^A – H] (*m/z* 164) in the negative ESI state because of the methoxy moiety on the B-structure, which indicated large differences between (R)-isosakuranetin and (R)-hesperetin. This work combined several levels to enhance the originality of structural annotation based on LC analysis combined with HRMS and produced vital outcomes in practice for the real detection of citrus flavanones (He et al., 2021). LC-HRMS and ^1^H NMR were used to determine the quality of table grapes during storage. In this study, three non-targeted procedures, LC-HRMS, ^1^H NMR, and HS-SPME/MS-eNose, together with chemometric analysis, were applied to group two table grape cultivars (Victoria and Italia) at five quality levels. Grapes in marketable quality states differ from those in nonmarketable quality conditions. PLS-DA and PCA-LDA were used, and the results indicated that MS-eNose produced the best results. In particular, with the table grapes in italia, mean prediction abilities from 98% to 99% and from 87% to 88% were produced for discrimination among the five quality states and non-marketability/marketability, respectively. For table grape cultivars from Vitoria, mean predictive abilities greater than 99% were observed in both groups. In addition, ideal models have been obtained for Victoria and Italia cultivars using HRMS and NMR data (Innamorato et al., 2020). Fig 2 illustrates the use of LC-HRMS to detect bioactive compounds in various fruits.

In an authentication study, UHPLC-HRMS phenolic profiling and multivariate calibration methods were utilized to characterize, classify, and authenticate fraud in cranberries (Barbosa et al., 2018). In this study, 53 polyphenolic standards were characterized to generate a user-accurate mass database, which was then used to extract the UHPLC and HRMS polyphenolic characteristics using the ExactFinder™ tool. Procyanidin A2, veratric, caffeic and sinapic acids are important polyphenols that distinguish cranberry-related products from other fruits (Barbosa et al., 2018). In a similar study, non-targeted UHPLC-HRMS to characterize, categorize, and authenticate cranberry- based pharmaceuticals and natural compounds (Barbosa et al., 2019). HRMS data in full-scan mode and at 70,000 full-width resolution were observed and analyzed using the MS conversion tool to generate peak intensity profiles as a function of retention periods and *m/z* values. Barbosa et al. (2019) employed a threshold peak filter for total intensity to reduce the complexity of the data. Partial least squares-discriminant and principal component analyses showed patterns used to analyze the estimated samples with regard to fruit origin, such as raspberry, blueberry, grape, and cranberry. In addition, the discrimination between cranberry-based pharmaceuticals and natural products has been observed. Both UHPLC-coupled HRMS fingerprints in positive and negative H-ESI states and data fusion of the two acquisition modes were shown to be ideal chemical analyzers for cranberry extract extraction. The authentication of the suggested methodology indicated a 100% sample prediction rate. The results were further subjected to partial least squares regression to identify frauds and estimate the percentage of fruit adulterants in cranberry fruit extracts, resulting in a prediction between 0.17-3.86 percent cents. They also proposed improvements or decreases in bioactive molecules due to the various extraction and purification modes applied by drug-producing industries during the preparation of raw extracts (Barbosa et al., 2019).

The chemical indices of the saskatoon berry fruits were analyzed using liquid chromatography. In addition, the free radical scavenging activities of the fruits and their constituents were estimated. The features of sugars and organic acids were estimated using HPLC, and the fruit-based constituents were analyzed using UHPLC. The antioxidant activity was estimated using the radical scavenging capacity procedure. The fruits of the investigated genotypes and their compounds differed markedly in their reactive radical quenching action and composition of sugars, organic acids, and biologically active molecules. Also, from the results, the ‘clone type S’ fruits displayed best reactive radical scavenging property (Lachowicz et al., 2019). Smoky’ and ‘Thiessen’ showed the highest polyphenol and organic acid content. Tartaric, quinic, and malic acid were also detected in these fruits. Cluster and principal component analyses aided in estimating the most vital variables and authenticating three classes of genotypes clustered based on similarities in the composition of individual molecular tests. Data from this study showed that saskatoon berry fruits and their constituents are important reservoirs of natural free radical scavenging compounds, whereas fruit parts, including seeds and peel, could be utilized in the development of novel functional and super foods with therapeutic actions, as well as pharmaceutical, cosmetic, or medical constituents (Lachowicz et al., 2019).

The solvent extract of *Styrax officinalis* fruit was analyzed using column chromatography, NMR, and MS analysis (Venditti et al., 2018)*.* They identified 15 molecules: demethylegonol, 1,2-di-α-linolenoyl-*sn*-glycerol, sucrose, 1,5-anhydro-D-mannitol, succinic acid, egonol, 1-α-linolenoyl-2-palmitoyl-*sn*-glycero, glutamic acid, 6'-O-benzoyl-sucrose, lactic acid, raffinose, homoegonol, 1,2-di-α-linolenoyl-3-linoleoyl-*sn*-glycerol, glucose, and tri-α-linolenoyl-*sn*-glycerol. The majority of these compounds are chemotaxonomically important. The confirmation of these compounds by the above approaches supported their ethnopharmacological applications (Venditti et al., 2018).

Chemical evaluation of volatile plant-based molecules from different fruit spirits that were homemade using headspace solid-phase microextraction was performed. In this study, 24 samples from seven types of fruit spirits (plum, pear, mirabelle, apricot, raspberry, apple, and cherry spirits) were assessed for volatile profiles (Bajer et al., 2017). Esters, particularly ethyl esters, were the predominant compounds. However, sesquiterpenes were found to be the most predominant type of volatile molecule by establishing the differences between stone and pome fruit spirits (by relatively higher levels of (*E,E*)-*α*-farnesene and content of alpha-zingiberene and (*E*)*-α*-bisabolene in pome fruit spirits volatile profiles). Ethyl salicylate and propyl decanoate have been detected only in stone fruit spirits. The work also stated that other molecules were discovered as being distinctive for individual types of estimated fruit spirits, including (*E*)*-β*-farnesene for apple, apricot spirits-based *γ*-decalactone, some apocarotenoids for raspberry spirits and (*Z*)-9-tetradecen-1-ol for mirabelle spirits (Bajer et al., 2017).

Chemometric and chemical methods have been used for profiling and analyzing small peptides and amino acids in different *Luo-Han-Guo* samples (Zhou et al., 2015). The data from this study presented three active small peptides and 24 free amino acids from *Luo-Han-Guo* in trace concentrations using hydrophilic interaction liquid chromatography (HILIC)-UHPLC-QTRAP®/MS^2^. *Luo-Han-Guo* contains high concentrations of essential amino acids, which are vital components of a healthy body system. The chemometric approaches of unsupervised PCA and supervised counter-propagation ANN (CP-ANN) were used to group and distinguish 40 groups of Luo-Han-Guo samples from various regions, types, and varieties. According to Zhou et al. (2015), the proposed approach can detect edible agricultural products and plants composed of small peptides and free amino acids.

The fruit-based polyphenol content was authenticated by HPLC and capillary zone electrophoresis (CZE). Navarro et al. (2014) used several procedures to maximise total polyphenol recovery or fractions, such as anthocyanins and phenolic acids. The data from HPLC and CZE were applied to PCA, which revealed that the samples were primarily clustered by the fruit of origin, with grape- and cranberry-based products separated into groups. HRMS was used to validate the presence of type A proanthocyanidins, which are commonly found in cranberry products (Navarro et al., 2014). Based on PCA interpretation, HRMS data revealed that the suspicious sample was not a cranberry-based product, validating and demonstrating the applicability of both HPLC- and CZE-suggested techniques for fruit-based product characterization (Navarro et al., 2014). Table 1 displays the metabolomic profiles of several fruits detected using mass spectrometry, where product ions are fragment ions generated from precursor ions during tandem mass spectrometry (MS/MS). These ions are detected by LC-MS or LC-HRMS analysis via collision-induced dissociation (CID), which breaks the precursor ions into smaller fragments, facilitating the identification and structural elucidation of the metabolites.

**Table 1:** Metabolomic profiles of various fruits identified using mass spectrometry.

No	Name of the Fruits	Analytical methods	Major bioactive compounds	Molecular ion (*m/z*)	Product ion (*m/z*)	Reference
1	Blueberry (*Vaccinium corymbosum* L.)	UPLC-DAD-QToF/MS and UPLC-Qtrap-MS/MS	Delphinidin 3-O-glucoside (mirtillin)	465	303	(Kim et al., 2025)
			Cyanidin 3-O-galactoside (ideain)	449	287	
			Cyanidin 3-O-glucoside (asterin)	449	287	
			Petunidin 3-O-galactoside	479	317	
			Cyanidin 3-O-arabinoside	419	287	
			Petunidin 3-O-glucoside	479	317	
			Peonidin 3-O-galactoside	463	301	
			Peonidin 3-O-glucoside	463	301	
			Malvidin 3-O-galactoside (primulin)	493	331	
			Peonidin 3-O-arabinoside	433	301	
			Malvidin 3-O-glucoside (enin)	493	331	
2	Bilberry, Rabbiteye, Elderberry, Black currant, Chokeberry	HPLC/PDA/ESI-MS	Cyanidin 3,5-diglucoside	611	449, 287	(Nakajima et al., 2004)
			Delphinidin 3-galactoside	465	303	
			Delphinidin 3-glucoside	465	303	
			Delphinidin 3-rutinoside	611	303	
			Cyanidin 3-galactoside	449	287	
			Delphinidin 3-arabinoside	435	303	
			Cyanidin 3-sambubioside	581	449, 287	
			Cyanidin 3-glucoside	449	287	
			Petunidin 3-galactoside	479	317	
			Cyanidin 3-rutinoside	595	287	
			Cyanidin 3-arabinoside	419	287	
			Petunidin 3-glucoside	479	317	
			Petunidin 3-rutinoside	625	317	
			Peonidin 3-galactoside	463	301	
			Malvidin 3-galactoside	493	331	
			Cyanidin 3-xyloside	419	287	
			Malvidin 3-glucoside	493	331	
			Malvidin 3-arabinoside	463	331	
3	*Punica granatum* L. cv. Ruby	LC-HRMS/MS	Gallic acid	169.01369	125	(Silva et al., 2021)
			(+)-Catechin	289.07176	179, 205, 245	
			(+)-Gallocatechin	305.06667	179	
			Ellagic acid	300.99899	229, 257, 284, 301, 302	
			Monogalloyl-hexoside	331.06706	125, 169, 211, 241, 271	
			Ellagic acid-pentoside	433.04124	300, 301	
			Ellagic acid-deoxyhexoside	447.05689	300, 301	
			Ellagic acid-hexoside	463.05181	300, 301, 302	
			Digalloyl-hexoside	483.07802	169, 193, 313, 331	
			HHDP Galloyl hexoside	633.07333	249, 275	
			Punicalin	781.05299	575, 601	
			Punicalagin α	1083.05926	275, 601, 781	
			Punicalagin β	1083.05926	275, 601, 781	
4	African Mango	UHPLC-HRMS	Ellagic acid	300.9978	301, 300, 284, 257, 229, 185	(Sun & Chen, 2012)
			Di-O-methyl-ellagic acid hexoside	491.0817	476, 328, 313	
			Methyl-ellagic acid	315.0136	300, 272, 244, 200	
			Galloyl-HHDP-ellagic acid	755.1072	711, 603, 453, 301, 284, 275	
			Kaemferol 3-O-glucoside	447.0919	357, 327, 301, 285, 284, 255	
			Quercetin 3-O-rhamnoside	447.0917	301, 300	
			Mono-O-methyl ellagic acid deoxyhexoside	461.0708	315, 300	
			Di-O-methyl ellagic acid	329.0290	314, 299, 271	
			Diosmetin	299.0549	284, 271, 227	
			Tri-*O*-methyl-ellagic acid	343.0449	328, 313, 298, 285	
			Galloyl-tri-O-methyl-ellagic acid hexoside	657.1071	343, 329, 328, 313, 285	
			Mono-O-methyl-ellagic acid rhamnoside	461.1074	315, 300	
5	Costa Rican apple	UPLC-DAD-ESI-MS/MS	Sinapic acid hexoside	385.1169	205, 223	(Navarro-Hoyos et al., 2021)
			Caffeoylquinic acid	353.0871	191, 179	
			p-Coumaroylquinic acid	337.0912	173	
			Shikimic acid	173.0447	93, 111	
			Feruloylquinic acid	367.0983	173, 191	
			Methyl-p-coumaroylquinic acid	351.1098	177	
			Feruloylquinic acid	367.0983	173, 191	
			Di-O-acetyl-O-p-coumaroylsucrose	571.1675	529, 553	
			Caffeoyl hexoside	341.084	161, 179	
			Phloridzin	435.1312	167, 273	
			Phloretin	273.0757	167, 201	
			Quercetin di-hexoside	625.1378	300, 301	
			Quercetin-rutinoside	609.1459	300, 301	
			Quercetin-pentoside	433.0732	300, 301	
			Quercetin acetyl hexoside	505.1002	300, 301	
			Quercetin-pentosylhexoside	595.1245	300, 301	
			Kaempferol-hexoside	447.0928	284.,285	
			Quercetin	301.0353	151, 179,	
			Procyanidin A-type dimer	591.1147	255, 273, 283, 289, 449,	
			Catechin	289.0708	205, 245, 271,	
			Procyanidin B-type dimer	577.1392	287, 289, 407, 425, 451, 559	
			Epicatechin	289.0708	205, 245, 271	
			(epi)catechin 3-O-gallate	609.1459	153, 289, 315	
			Vomifoliol-pentosilhexoside	517.2293	205, 385	
6	Blueberry, Raspberry, Blackberry, Cranberry and Cherry	UHPLH-HRMS	Afzelin	431.0984	285.0401	(Kodikara et al., 2024)
			Apigenin	269.0456	225.0551, 201.0551, 149.0239	
			Arbutin	271.0823	161.0452, 203.9364	
			Aromadendrin	287.0561	259.0608, 243.0659	
			Caffeic acid	179.035	135.0449	
			Caftaric acid	311.0409	179.0344, 149.0086	
			(+)-Catechin	289.0718	245.0811, 205.0499, 173.4917, 179.0344	
			Chlorogenic acid	353.0878	191.0559	
			Cyanidin	285.040	246.9308, 213.0542, 259.0598	
			Cyanidin-3-arabinoside	419.0973	284.0325	
			Cyanidin-3-glucoside	449.1078	287.0547	
			Cyanidin-3-rutinoside	595.1658	287.054	
			Daidzein	253.0506	227.0698, 199.0748	
			Daidzin	415.1035	255.0647, 338.3414	
			Delphinidin	303.0499	257.044, 229.0492	
			Ellagic acid	300.999	257.0089, 229.014, 185.0242	
			(-)Epicatechin	289.0718	245.0815, 205.0502	
			(-)-Epicatechin gallate	441.0827	289.0712, 169.0318	
			(-)-Epigallocatechin	305.0667	179.0347, 221.0453	
			Ferulic acid	193.0506	149.0603	
			Fisetin	285.0405	163.0034, 135.0085, 257.0452, 241.0503	
			Gallic acid	169.0143	125.0241	
			Genistein	269.0456	159.0446, 133.0291, 225.0551, 241.0503	
			Glycitein	283.0606	351.0483	
			Glycitin	445.114	283.0612, 282.053	
			Herbacetin	301.0354	272.0279	
			Hesperetin	301.0718	286.0483	
			Isoquercetin	463.0882	301.0346	
			Isorhamnetin	315.051	315.0513, 300.0278	
			Isovitexin	431.0984	311.0556, 341.0659	
			Kaempferol	285.0405	151.0034, 257.0452	
			Kaempferol-3-glucoside	447.0933	284.0323, 285.0402, 327.0808	
			Liquiritingenin	255.0663	135.0086	
			Luteolin	285.0405	241.0499, 199.0895, 175.0395, 157.0031	
			Malvidin	331.0819	315.0499, 316.0579, 299.0552, 287.0552	
			Malvin	655.1854	493.1328, 331.0802	
			Myricetin	317.0303	178.9982, 151.0033	
			Naringin	579.1719	459.114	
			Nicotiflorin	593.1512	285.0397	
			Orientin	447.0933	327.0506, 357.061	
			Okanin	287.0561	135.0440	
			Pelargonidin	271.0594	193.1306	
			Peonidin	301.0712	286.0468, 284.0321	
			Petunidin	317.0655	302.042	
			Piceatannol	243.0663	225.0557, 201.0556	
			Polydatin	389.1242	227.0712	
			Procyanidin A2	575.1195	423.0718, 449.0873, 289.0712	
			Procyanidin B2	577.1352	425.0878, 407.0765, 289.0712	
			Procyanidin C2	865.1985	695.1401, 577.1348, 739.166, 713.166	
			Protocatechuic acid	153.0193	109.0291	
			Quercetin	301.0354	151.0033	
			Resveratrol	227.0714	185.0605	
			Rhapontin	419.1348	257.0817	
			Rutin	609.1461	301.0349	
			Sinapic acid	223.0612	179.071, 164.0475	
			Taxifolin	303.051	285.04	
			Vanillic acid	167.035	123.0446	
			Vicenin-2	593.1512	473.1082, 503.1188, 353.066	
			Vitexin	431.0984	311.0552	
7	*Rhodomyrtus* *tomentosa* fruits	UHPLC-MS	Quinic acid	191.0557	85.0288, 87.0080, 101.0232, 111.0082, 129.0190, 155.0000, 173.0096	(Divya Priya & Martin, 2025)
			Gallic acid	169.0144	169.0144, 125.0239, 124.6283	
			Pyrogallol	125.0239	125.0239, 107.0138, 97.0294, 81.0347, 69.0339, 79.01891	
			Chlorogenic acid isomer	353.0758	353.0725, 317.0415, 264.0225, 173.0082, 111.0091	
8	Goji berry *Lycium barbarum*	UPLC–QTOF/MS	Ferulic acid di-hexose	649.2007	649.2015, 566.2046, 487.1454, 325.0925, 229.0509, 163.0395, 89.0245	(Bondia-Pons et al., 2014)
			Coumaric acid hexose	325.0924	371.0984, 325.0937, 229.0528, 163.0395, 145.0274, 119.0499	
			Caffeic acid	179.0341	179.0341, 135.0454, 107.0500, 89.0245, 59.0144	
			Kaempferol dihexose deoxyhexose	755.2043	755.2062, 711.2656, 616.3801, 591.2050, 465.1007, 354.1152, 300.0252, 242.6408, 165.0433, 312.1236	
			Lyciumide A	312.1244	297.0979, 190.0506, 178.0503, 148.0524, 135.0452	
			Myristic acid	227.2014	227.2014	
			Palmitic acid	255.2322	255.2322	
9	Goji berry *Lycium*	LC-ESI-MS/MS	Rutin	609	255, 271, 300	(Jarouche et al., 2019)
			Narcissin	623	299, 315	
			Nictoflorin	593	255, 284	
			Coumaric acid	164	93, 120	
			Scopoletin	191	103, 176	
			Caffeic acid	179	135	
			Chlorogenic acid	353	93, 191	

## 6 Application of LC-HRMS in foodomics and nutraceutical research: A focus on fruits

LC-HRMS has become an important method in foodomics, providing a full depiction of the complicated chemical composition of fruits and their health and nutritional benefits (Beteinakis et al., 2024; Mahato et al., 2024). Berries and citrus and tropical fruits have been thoroughly investigated because of their high concentrations of polyphenols, flavonoids, vitamins, and other bioactive components (Romero-Martínez et al., 2025). In berries, LC-HRMS allows for the identification of anthocyanins, ellagitannins, and flavonols, all of which contribute to their antioxidant and anti-inflammatory properties (Rai & Tzima, 2021). For instance, a complete metabolomic investigation of blueberries and blackberries showed substantial interspecies variation in anthocyanin concentrations, allowing cultivars with higher nutraceutical potential to be selected (Wu et al., 2022). Strawberry LC-HRMS fingerprinting has also been used to track changes in polyphenolic chemicals during ripening and storage, correlating metabolite changes with sensory quality and shelf life (La Barbera et al., 2017). LC-HRMS detected important flavanones (e.g., hesperidin and naringin) and limonoids in citrus fruits, which have been linked to cardiovascular and anti-obesity effects (Sanches et al., 2022). Recent studies employing Orbitrap-based LC-HRMS have succeeded in mapping the citrus metabolome, revealing biomarkers linked to fruit development and variation among varieties (Wang et al., 2024a). Tropical fruits, including mangoes, guava, and papaya, benefit from the LC-HRMS analysis. A study on mango cultivars using Q-TOF LC-HRMS observed distinct terpenoids and gallotannins associated with antioxidant capacity and consumer preferences (Aydoğan, 2020). Similarly, an LC-HRMS study on guava revealed novel phenolic glycosides with antibacterial characteristics, indicating their potential utility in functional food production (Lorena et al., 2022).

Authentication and traceability are essential to maintain food quality, safety, and consumer confidence. LC-HRMS can detect adulteration and verify the authenticity of fruits and their derivative products (e.g., juices, powders, and extracts) by detailed metabolite profiling (Medina et al., 2019). Food authentication using LC-HRMS entails comparing metabolomic profiles to reference databases to assure species or varietal identification. For example, LC-HRMS has been used to identify wild bilberries and blueberries, which are occasionally illegally swapped for nutraceutical products owing to differences in price (Salo et al., 2021). Small differences in the flavonoid composition in citrus have been exploited to identify the regional origin and cultivar type (Cetinkaya et al., 2025). Another important step is the detection of adulteration (Thiruvengadam et al., 2025). LC-HRMS has been used to detect the presence of low-cost fillers such as apple or pear juice in pomegranate and grape juice. Indigenous metabolites can be identified and quantified, enabling a more accurate detection of food fraud (Dasenaki & Thomaidis, 2019). Furthermore, by employing isotope ratio analysis and multivariate chemometric models with LC-HRMS data, the fruit products can be tracked along the supply chain. This method not only ensures origin verification, but also encourages adherence to food safety and labelling guidelines. In summary, LC-HRMS has revolutionized foodomics by enabling high-throughput, sensitive, and precise profiling of fruit metabolites. Their applications in nutraceutical research, authenticity, and food quality make them indispensable tools in modern food science.

## 7 Integration of LC-HRMS with other omics techniques

The use of LC-HRMS-based metabolomics and other omics technologies, such as transcriptomics, genomics, and proteomics, has altered the system-level recognition of complex biological processes in fruits, particularly for the identification and functional validation of bioactive substances (Chele et al., 2025). Each omics layer generates a unique image of cellular activity. Genomics depicts the genetic potential, transcriptomics portrays gene expression fluctuations, proteomics represents protein quantity and alterations, and metabolomics records the byproducts of biological activity. Genomic data can be used in fruit research to identify the genes involved in secondary metabolite biosynthesis. Researchers can link metabolic variables to genetic loci by merging LC-HRMS data with genomic and transcriptome information using genome-wide association studies (GWAS) or quantitative trait locus (QTL) mapping (Allwood et al., 2021). For example, metabolite-gene correlation investigations in grapes have identified transcription factors (e.g., *MYB* and *bHLH*) that regulate anthocyanin and flavonol production (Niu et al., 2025).

Transcriptomic integration has been beneficial for dynamic investigations of fruit development, ripening, and stress responses. In tomatoes, combining RNA-seq with LC-HRMS metabolomics has revealed an integrated transcriptional-metabolic network that regulates carotenoid formation and flavor volatile synthesis throughout ripening (Bai et al., 2023). Similarly, citrus transcriptome-metabolome studies have revealed the regulatory mechanisms involved in terpenoid and flavonoid synthesis under biotic stress (Zhao et al., 2024). Proteomics, especially when paired with metabolomics, can reveal the mechanistic details of enzyme control and metabolic flux. Label-free quantitative proteomics during banana fruit ripening, combined with LC-HRMS sugar and polyphenol profiling, provides a comprehensive overview of ethylene signalling and metabolic changes (Yin et al., 2022). These multi-omics approaches allow researchers to detect metabolic changes specific to time and tissue.

**Fig 3:**
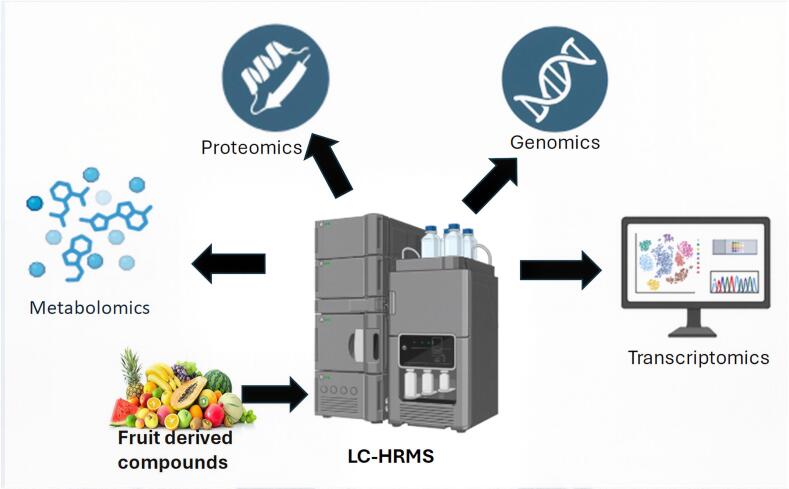
Integration of LC-HRMS with multi-omics in fruit-based computational biology.

Systems biology employs multi-omics integration to reassemble biological systems, thereby enabling the discovery of novel fruit-derived bioactive compounds with nutraceutical properties. LC-HRMS is important because it provides high-resolution metabolic phenotypes (metabotypes) that can be linked to genetic and proteomic profiles (Erny et al., 2020). For example, a systems biology study of strawberries used metabolomic, transcriptomic, and proteomic data to map the flavonoid biosynthetic pathway and identify new glycosyltransferases responsible for anthocyanin diversity, which are important for both nutritional quality and consumer appeal (Guo et al., 2023). This strategy promotes functional gene identification and the metabolic engineering of fruit crops. Furthermore, system-based modelling, such as metabolic flow analysis and network topology modelling, can predict metabolite accumulation trends and pinpoint control nodes for route optimization. In kiwifruit, combined omics and flux balance analyses projected higher vitamin C production under various stress conditions, leading to nutritional content-boosting techniques (Ren et al., 2024). Furthermore, systems biology helps identify the synergistic bioactivity of fruit components in nutraceutical research. For example, integrating LC-HRMS metabolomics with transcriptomics demonstrated that ellagitannins and flavonols in berries work together to influence inflammation-related gene expression in human cell lines (Gutierrez et al., 2017). Fig 3 shows the integration of LC-HRMS with multiomics in fruit-based computational biology.

## 8 Advantages and disadvantages of other techniques compared to LC-HRMS

LC-HRMS offers several advantages over GC-MS and other analytical techniques, particularly for the analysis of complex biological, environmental, and plant samples. LC-HRMS can directly analyze nonvolatile, thermally labile, and highly polar metabolites (such as amino acids, sugars, phenolics, and glycosides) without derivatization, unlike GC-MS, which requires analytes to be volatile. This feature enables LC-HRMS to cover a much wider range of metabolites (Ren et al., 2018). LC-HRMS instruments, particularly those with Orbitrap or TOF analyzers, provide a very high mass accuracy (within a few ppm) and resolving power. This allows for differentiation between isobaric compounds and the confident identification of molecular formulas (Stettin et al., 2020). As it does not include derivatization, LC-HRMS requires fewer sample preparation procedures. GC-MS derivatization is time-consuming, introduces artifacts, and destroys heat-sensitive compounds. LC-HRMS maintains native chemicals while reducing analytical errors (Stettin et al., 2020). LC-HRMS is useful for untargeted metabolomics, biomarker discovery, and novel molecule identification because of its ability to detect unknown compounds in the absence of existing reference spectra. Although GC-MS excels in identifying known volatile compounds, it has drawbacks when dealing with unknown nonvolatile molecules (Wu et al., 2012). Furthermore, LC-HRMS is highly sensitive to chemicals such as phenolics, alkaloids, glycosides, peptides, and lipids, which ionize readily under ESI. These compounds are frequently difficult to analyze by GC-MS. LC-HRMS employs soft ionization techniques (e.g., ESI and APCI) to generate intact molecule ions with minimal fragmentation. This facilitates structural elucidation and accurate mass determination, unlike GC-MS, which often employs harder electron ionization (Stettin et al., 2020).

Although LC-HRMS provides superior mass accuracy and metabolite coverage, GC-MS and other analytical techniques offer distinct features that make it essential in analytical chemistry. GC-MS uses hard ionization procedures, such as electron impact or chemical ionization, which create repeatable fragmentation patterns that are ideal for compound identification. Standardized spectra are well represented in large libraries, such as NIST and Wiley, allowing for the accurate identification of known volatile compounds. In contrast, LC-HRMS generates soft fragmentation spectra that are less standardized and more difficult to interpret (Arruda et al., 2023; Wu et al., 2012). GC-MS is more suited for tiny, volatile, or thermally stable compounds, such as environmental contaminants, insecticides, flavor and fragrance molecules, and hydrocarbons. This approach provides better chromatographic separation, less matrix interference, and a higher sensitivity for certain analytes than LC-HRMS (McNair et al., 2019). GC-MS has a significant advantage in terms of spectrum database resources. Its vast EI-based spectrum libraries enable the rapid and consistent identification of known analytes, eliminating the need for unique standards for each compound. LC-HRMS lacks extensive spectral libraries for many non-volatile or polar compounds; hence, GC-MS is the preferred method for identifying known volatiles (Arruda et al., 2023; McNair et al., 2019). GC-MS devices are often cheaper to operate and maintain than are LC-HRMS systems. They are simpler to prepare, have fewer ion suppression difficulties, and do not require high-purity solvents or sophisticated desolvation systems. Thus, GC-MS is appropriate for routine volatile compound analyses in environmental, forensic, and food laboratories (Arruda et al., 2023; McNair et al., 2019). The highly consistent fragmentation patterns in GC-MS, along with minimal matrix effects, enable the exact quantification of volatile substances, such as flavor components, steroid volatiles, and residual solvents. GC-MS typically has lower detection limits for these compounds than LC-HRMS (Arruda et al., 2023; McNair et al., 2019). Owing to variations in volatility and polarity, GC columns frequently produce superior isomeric compound resolutions compared to LC columns. Thermal diffusion and phase interactions in GC enable unambiguous separation of closely related isomers, which the LC may co-elute. Overall, LC-HRMS outperformed GC-MS in adaptability, compound coverage, sensitivity, and structure. This is especially beneficial in metabolomic studies, plant extract profiling, and pharmacokinetic studies, where complex combinations of thermally labile and polar compounds are present. In contrast, GC-MS and other analytical procedures surpass LC-HRMS for the analysis of volatile, semi-volatile, and thermally stable substances. They provide excellent consistency, stronger spectrum libraries, increased sensitivity for appropriate analytes, and simpler and more cost-effective procedures. GC-MS is an ideal instrument for environmental, forensic, and food chemistry applications involving volatile compounds, owing to its advantages.

## 9 Challenges and opportunities

Despite the lack of full statistical analysis, targeted analytical processes provide critical information about the presence and concentration of crucial variables in a sample. However, quantitative and qualitative estimations are frequently constrained because they require expensive analytical standards and additional quantitative methods, with significant levels of chemical consumption. Furthermore, appropriate extraction techniques for specific analytes that require maximal recovery and good HPLC separation times are required. Achieving adequate peak resolution and optimization makes targeted methods labor-intensive, costly, and multi-stepped (Klikarová & Česlová, 2022). Although advances in technology have enhanced the detection of fruit adulteration through highly sensitive chemical and biological analyses, targeted methods are still limited because they cannot detect all possible forms of counterfeiting. Non-targeted methods, such as sample profiling or fingerprinting, do not aim to identify or quantify individual analytes. Instead, they analyze the overall sample patterns, which eliminates the requirement for analytical standards or quantitative calibration procedures such as multi-standard additions, calibration curves, or direct comparison. Non-targeted procedures employ different extraction and separation optimization strategies than the focused methods. The goal was to obtain multiple distinct peaks in the chromatogram that represented a wide spectrum of metabolites. The resolution of these peaks is determined by the physicochemical qualities of the metabolites as well as the analytical methodology used for extraction, separation, and detection. Because non-targeted studies need no sample preparation, calibration solutions, and measurement of individual peaks, they save time and money while reducing operator error. Although the final stage of statistical analysis remains time-consuming, the application of multivariate statistical techniques allows for a more thorough interpretation of sample profiles than standard targeted approaches (Klikarová & Česlová, 2022).

## 10 Conclusion

Chemical profiles, both targeted and non-targeted, provide essential insights into plant structure and allow high-throughput data collection of different phytochemicals. As stated in this review, techniques include UHPLC, GC-MS, and HRMS. These analytical approaches provide a wide range of chemical information; however, they require advanced instrumentation and specialized knowledge to ensure a full profile of compounds with varying physicochemical features. The insights provided by chemical profiling have numerous uses in agriculture, food science, and pharmaceuticals. The discovery of bioactive chemicals has aided agricultural improvement initiatives, improved postharvest storage, and accelerated the development of functional foods and natural therapies. Profiling is useful for quality control, detection of adulterants, and verification of product authenticity. Future improvements in chemical profiling are needed to overcome the current issues and open up new opportunities. Integration with omics technologies, such as metabolomics, genomics, and proteomics, shows promise in unraveling complicated plant biochemical networks. Combined with the development of machine learning algorithms and automated data analysis platforms, these methods will improve the speed, precision, and scalability of plant metabolite characterization. Machine learning methods, for example, can be used to predict metabolite functions, improve data integration, and discover novel bioactive compounds. Refined chemical profiling technologies have practical uses such as developing sustainable farming practices, precisely breeding nutrient-dense crops, and providing personalized nutritional solutions based on individual health profiles. Advances in bioactive chemical discovery could transform precision medicine by delivering plant-based therapies targeting specific disease pathways. The convergence of chemical profiling with multi-omics approaches, real-time analytics, and artificial intelligence will likely redefine the landscape of plant science and its applications, paving the way for innovations in food security, natural product development, and global healthcare.

## CRediT authorship contribution statement

**Abdur Rauf:** Writing – original draft, Methodology. **Ahmed Olatunde:** Data curation, Writing – review & editing. **Zuneera Akram:** Writing – review & editing. **Hassan A. Hemeg:** Writing – original draft. **Anees Ahmed Khalil:** Conceptualization, Methodology, Data curation, Writing – original draft, Writing – review & editing. **Mohammad Ali Shariati:** Software, Methodology. **Zehra Edis:** Writing – review & editing. **Muthu Thiruvengadam:** Writing – original draft, Validation, Methodology. **Seung-Hyun Kim:** Writing – review & editing, Validation, Supervision, Investigation.

## Funding

This work was supported by a 10.13039/501100003725National Research Foundation of Korea (NRF) grant funded by the Korean government (MSIT) (RS-2025-00557207).

## Declaration of competing interest

The authors declare that they have no competing financial interests or personal relationships that could influence the work reported in this study.

## Data Availability

No data was used for the research described in the article.
